# Genetic variants alter T-bet binding and gene expression in mucosal inflammatory disease

**DOI:** 10.1371/journal.pgen.1006587

**Published:** 2017-02-10

**Authors:** Katrina Soderquest, Arnulf Hertweck, Claudia Giambartolomei, Stephen Henderson, Rami Mohamed, Rimma Goldberg, Esperanza Perucha, Lude Franke, Javier Herrero, Vincent Plagnol, Richard G. Jenner, Graham M. Lord

**Affiliations:** 1 Department of Experimental Immunobiology, King’s College London, London, United Kingdom; 2 NIHR Biomedical Research Centre at Guy’s and St Thomas’ Hospital and King’s College London, London, United Kingdom; 3 UCL Cancer Institute, University College London, London, United Kingdom; 4 UCL Genetics Institute, University College London, London, United Kingdom; 5 The Francis Crick Institute, London, United Kingdom; 6 Department of Genetics, University Medical Center Groningen, University of Groningen, Groningen, The Netherlands; The University of Queensland, AUSTRALIA

## Abstract

The polarization of CD4+ T cells into distinct T helper cell lineages is essential for protective immunity against infection, but aberrant T cell polarization can cause autoimmunity. The transcription factor T-bet (TBX21) specifies the Th1 lineage and represses alternative T cell fates. Genome-wide association studies have identified single nucleotide polymorphisms (SNPs) that may be causative for autoimmune diseases. The majority of these polymorphisms are located within non-coding distal regulatory elements. It is considered that these genetic variants contribute to disease by altering the binding of regulatory proteins and thus gene expression, but whether these variants alter the binding of lineage-specifying transcription factors has not been determined. Here, we show that SNPs associated with the mucosal inflammatory diseases Crohn’s disease, ulcerative colitis (UC) and celiac disease, but not rheumatoid arthritis or psoriasis, are enriched at T-bet binding sites. Furthermore, we identify disease-associated variants that alter T-bet binding *in vitro* and *in vivo*. ChIP-seq for T-bet in individuals heterozygous for the celiac disease-associated SNPs rs1465321 and rs2058622 and the IBD-associated SNPs rs1551398 and rs1551399, reveals decreased binding to the minor disease-associated alleles. Furthermore, we show that rs1465321 is an expression quantitative trait locus (eQTL) for the neighboring gene *IL18RAP*, with decreased T-bet binding associated with decreased expression of this gene. These results suggest that genetic polymorphisms may predispose individuals to mucosal autoimmune disease through alterations in T-bet binding. Other disease-associated variants may similarly act by modulating the binding of lineage-specifying transcription factors in a tissue-selective and disease-specific manner.

## Author summary

Research to date has identified many genetic variants that are more common in people with a particular disease. However, in conditions that reflect multiple genetic and environmental factors, it is difficult to know with certainty if and why any particular genetic variant is causative and the mechanism that may underlie this. Such variants are often outside of protein-coding exons, instead falling in regions that regulate gene expression. In these cases, the genetic variation may alter transcription factor binding and subsequent gene expression. In this study, we have examined how genetic variation affects T-bet binding to DNA, as a key transcriptional regulatory mechanism in the immune response. An inability to mount this response effectively can result in increased susceptibility to infections or cancer, while a response that is too strong, or wrongly targeted, can result in uncontrolled/chronic inflammatory and autoimmune conditions. We have found that T-bet binding sites are specifically enriched in genetic variants associated with the mucosal autoinflammatory diseases UC, Crohn’s disease and celiac disease. We also identify genetic variants that alter T-bet binding and gene expression. This discovery thus identifies a molecular mechanism through which genetic variants can be associated with increased risk of mucosal autoimmune disease.

## Introduction

The differentiation of naïve CD4+ T cells into distinct T helper cell (Th) lineages is essential for adaptive immunity. The original paradigm of interferon-gamma (IFN-γ) producing T-helper 1 (Th1), and type-2 (Interleukin 4, 5, and 13) cytokine producing Th2 cells has expanded to include both Interleukin-17 (IL-17) producing Th17 and anti-inflammatory T-regulatory (Treg) cells. Th cell differentiation is controlled by a set of master regulatory or lineage-specifying transcription factors, with the T-box family member T-bet necessary and sufficient for Th1 cell differentiation. GATA3, ROR*γ*T and FOXP3 perform parallel roles in Th2, Th17 and Treg cells, respectively [1]. Importantly, T-bet inhibits alternative lineage fate specification, repressing both the Th17 and Th2 lineages [2–4].

Inappropriate Th cell activation and polarization can lead to autoimmunity. Worldwide, autoimmune and auto-inflammatory diseases are now estimated to affect nearly 10% of the population [5]. The incidence of inflammatory bowel diseases (IBD), including Crohn’s disease and UC, and celiac disease, is rising rapidly, with more than 1.4 million people affected in the USA alone [6]. A role for T-bet is particularly apparent in the mucosal immune system and has been linked to IBD and celiac disease [7]. The expression of T-bet is upregulated in lamina propria T cells of patients with Crohn’s and celiac disease and e*x vivo* culture of biopsies from untreated celiac patients with gliadin increases T-bet expression through STAT1 activation [8,9]. In addition to this, it is now apparent that mucosal inflammation is also driven by IL-17, which is enhanced by IL-23 receptor signals in effector T cells [10]. Loss of T-bet in the innate immune system leads to a transmissible form of ulcerative colitis in the TRUC (T-bet and Rag deficient Ulcerative Colitis) model, driven by transcriptional derepression of *TNF* in colonic mononuclear phagocytes [11–13]. This susceptibility has also been shown to be dependent on IL-17 and mediated via repression of IL-7 receptor expression by T-bet in innate lymphoid cells (ILCs) [11]. T-bet has subsequently been shown to play a role in the development of the NKp46^+^ CCR6^-^ subset of IL-22 expressing ILCs that, in turn, are important for protecting the epithelial barrier during *Salmonella enterica* infection [14,15].

Autoimmune diseases cluster in families, suggesting a large genetic component [16]. Genome-wide association studies (GWAS) have identified hundreds of risk loci for autoimmune diseases, including for IBD and celiac disease [16–23]. The majority of autoimmune disease-associated SNPs lie outside of gene coding regions in intergenic or intronic regions [24]. This can make it challenging to understand the molecular basis of how a genetic variant predisposes to disease. Furthermore, the causal variant can be difficult to identify from the large clusters of SNPs in linkage disequilibrium that tend to be identified by GWAS. Thus, efforts have been made to identify SNPs located within regulatory elements marked by open chromatin, histone modifications associated with active enhancers or known/predicted transcription factor binding sites [21,24–32]. Some of these variants have been shown to modulate transcription factor binding or epigenetic regulation. Genetic variants that alter DNase I hypersensitivity [27,33,34], DNA methylation [35–38], histone modification [27,39–43], and the binding of transcriptional regulators to DNA [27,33,34,44–51], have been identified, suggesting potential causal mechanisms.

Although previous studies have demonstrated enrichment of transcription factor binding sites at disease-associated polymorphisms, whether specific disease causing variants act to alter the binding of T cell lineage-specifying factors has not been investigated. Having previously mapped T-bet binding across the genome in human Th1 cells [52–54] we used a systematic functional GWAS (fGWAS) approach to determine the degree to which disease-associated SNPs were enriched within T-bet binding sites. SNPs were then tested for effects on T-bet binding *in vitro* using a novel flow cytometric assay and *in vivo* by allele-specific ChIP-seq. We report here that SNPs associated with mucosal inflammatory diseases are selectively enriched at T-bet binding sites. Furthermore, we show that the celiac disease associated variants of rs1465321 and rs2058622, and the IBD-associated variants of rs1551398 and rs1551399, exhibit decreased T-bet binding *in vivo*. We further demonstrate that the genes associated with these SNPs, *IL18RAP* and *TRIB1*, respectively, are transcriptionally regulated by T-bet and that rs1465321 is an expression quantitative trait locus (eQTL) for *IL18RAP*. Taken together, these data mechanistically link alterations in T-bet binding to disease predisposition.

## Results

### Disease-associated SNPs at T-bet binding sites

**Fig 1:**
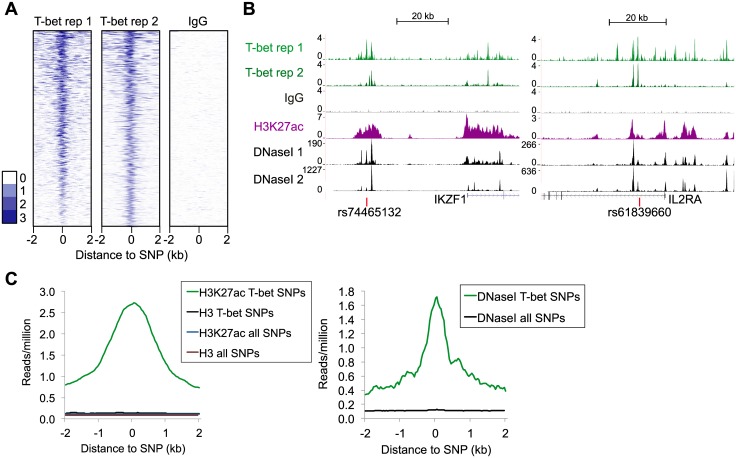
T-bet binding at polymorphic sites. **A.** Heat map showing T-bet occupancy around SNPs located within T-bet binding sites (T-bet hit-SNPs). Each row is centred on a single SNP, with T-bet binding shown across the genomic region stretching 2 kb up and downstream. Sequence reads (per million total reads) at each position are represented by colour, according to the scale on the left. Negative IgG ChIP-seq data are shown on the right at the same loci. **B.** T-bet binding at two example T-bet hit-SNPs. The number of sequencing reads from T-bet, IgG control and H3K27ac ChIP-enriched DNA are plotted per million input-subtracted total reads and aligned with the human genome. DNaseI hypersensitivity data (2 replicates) are from ENCODE. **C.** Left: Average number of ChIP-seq reads for H3K27ac and control total H3 in human Th1 cells plotted against the genomic distance from T-bet hit-SNPs or the complete set of GWAS SNPs plus those in high LD. Right: Average number of sequencing reads measuring DNaseI hypersensitivity plotted against genomic distance.

To identify disease-associated polymorphisms at T-bet binding sites, we compared the locations of GWAS hits listed in the National Human Genome Research Institute (NHGRI) catalogue [55] with binding sites for T-bet in primary human Th1 cells [52–54]. As the published trait-associated SNP may not be the most functionally relevant [28], SNPs in high linkage disequilibrium LD (r^2^ >0.8) were also examined. This returned a list of 926 unique SNPs located at T-bet binding sites (hereafter referred to as T-bet hit-SNPs; Fig 1A and 1B, S1A Fig and S1 Table). In line with previous reports, a minority (143) of the T-bet hit-SNPs were the putative causal SNP from GWAS data, with the others being in high LD (total of 621 independent LD blocks). Examination of the location of T-bet hit-SNPs in relation to protein-coding genes revealed that the majority (63%) were distal (>1 kb) to gene promoters. As expected, H3K27ac and DNaseI hypersensitivity were highly enriched in Th1 cells at T-bet hit-SNPs compared with all disease-associated SNPs, consistent with these being located within active regulatory elements (Fig 1C).

### SNPs associated with mucosal immune diseases are enriched at T-bet binding sites

As T-bet is only expressed in cells of the immune system, we hypothesised that T-bet hit-SNPs would be primarily associated with autoimmune diseases. To test this, we used fGWAS [56], a hierarchical model that assesses relative enrichment of GWAS associations within various functional elements. This model splits the genome into large blocks (larger than regions of linkage disequilibrium), assesses whether each block contains a SNP associated with the trait of interest or not and then searches among supplied functional annotations for those that improve the likelihood of predicting the presence of a trait-associated SNP, finally predicting which SNP in the block is most likely causal.

**Fig 2:**
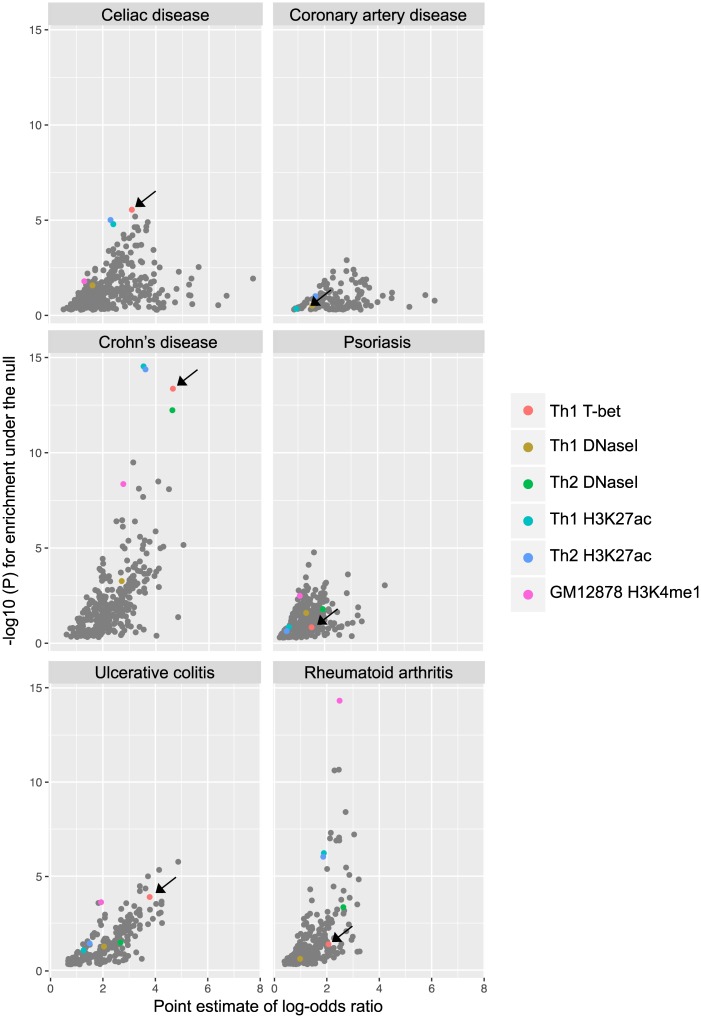
SNPs associated with mucosal autoimmune diseases are enriched at T-bet binding sites. Scatter plots showing log-odds ratio against –log(10) p-value for the enrichment of disease-associated SNPs at different functional annotation datasets (DHS, histone modification, FAIRE-seq and transcription factor binding). Selected enriched functional annotation datasets are highlighted. GM12878 H3K4me1 indicates sites of H3K4me1 in the GM12878 lymphoblastoid cell line. Celiac disease, Crohn’s disease and UC-associated SNPs, but not RA, psoriasis or coronary artery disease-associated SNPs, are strongly enriched at T-bet binding sites (red dots with arrows).

To test whether disease-associated SNPs were enriched at T-bet binding sites, we gathered GWAS data for the Th1-associated auto-inflammatory conditions celiac disease, Crohn’s disease, UC, rheumatoid arthritis (RA), psoriasis and, as a negative non-immune control, coronary artery disease (Fig 2). We compared T-bet binding sites with a number of other relevant functional annotations, including Th1 and Th2 cell DHS [57], H3K27ac [58], and sites of histone modification and transcription factor binding in immune cell lines from the ENCODE project [26] and other sources (S2 Table). Notably, we found that SNPs associated with all of the mucosal immune-mediated diseases tested (Crohn’s disease, UC and celiac disease) were enriched at T-bet binding sites, with the effect in Crohn’s disease being particularly striking. Enrichment at T-bet binding sites was similar to, or stronger than, DHS and H3K27ac and, in the case of Crohn’s and celiac disease, stronger than any other sets of transcription factor binding sites. As expected, SNPs associated with coronary artery disease were not enriched at T-bet binding sites. Of interest, no enrichment for T-bet binding sites was observed for RA- or psoriasis-associated SNPs, suggesting a specific role for altered T-bet binding in mucosal inflammatory disease.

To confirm that T-bet binding is enriched at IBD-associated SNPs, we compared T-bet binding sites to a set of credible SNPs identified at 94 IBD-associated loci [21]. We found that T-bet binding sites were more highly associated with credible SNPs than other SNPs at the same loci (93 bound by T-bet, p = 1.4x10^-5^, Fisher exact test). Furthermore, within the set of credible SNPs, the higher the posterior probability for causality, the more likely that the SNP overlapped a T-bet binding site (p = 6.3x10^-6^, continuous binomial regression, S1B Fig). The association of T-bet binding with causal SNPs is highlighted by the finding that, of the 93 credible SNPs bound by T-bet, 11 are the lead variants for their loci. Three of these (rs74465132, rs1887428 and rs61839660) have a posterior probability for causality of greater than 95%. These data suggest that the strong association of these SNPs with IBD is related to T-bet binding at these sites.

### Detection of altered T-bet binding at disease-associated variants by OligoFlow

**Fig 3:**
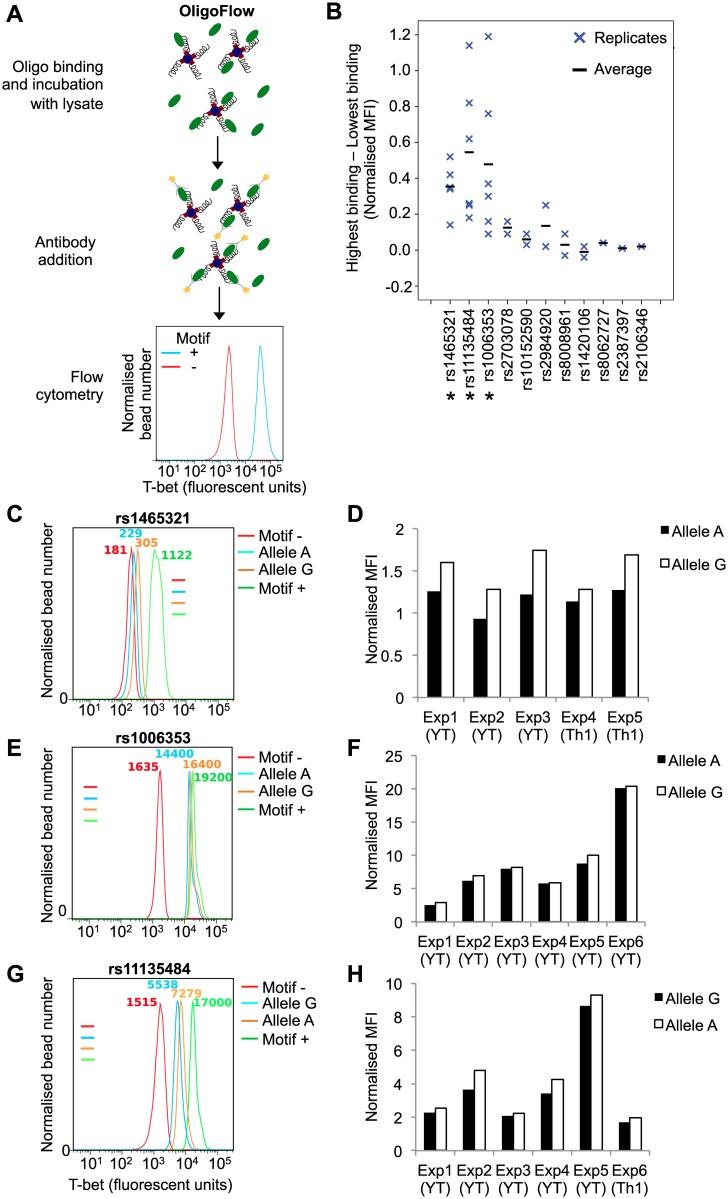
Genetic variants alter T-bet binding *in vitro*. **A.** Outline of the OligoFlow method. Double-stranded oligonucleotides are annealed to beads and incubated with cell lysate containing the transcription factor of interest. Fluorescently labelled antibody is added and MFI of the beads measured by flow cytometry. The histograms show the MFI of beads coated with oligonucleotides containing a T-bet binding motif (Motif +) or a mutated sequence (Motif -) after incubation with YT lysate, normalised for the number of beads acquired. **B.** Summary of OligoFlow results for the 11 SNPs tested. In each case, MFI for both alleles is normalised such that the negative control equals 1. Normalised MFI for the lowest binding allele was then subtracted from the value for the highest binding allele. Each cross represents one experiment, with the average difference between alleles represented by a horizontal line. * Significantly different binding between the two alleles (p < 0.05, paired t-test.) **C.** Representative experiment measuring the binding of T-bet to the A and G alleles of rs1465321. Data for the different oligonucleotide probes are separated according to the key on the right and the MFI is also shown. **D.** Bar chart showing all replicate experiments for rs1465321. The y-axis shows MFI for each allele normalised to the MFI of the negative control oligonucleotide (set to 1). Each pair of bars represents one experiment, performed with either YT cells (YT) or Th1-polarised primary CD4^+^ cells (Th1). **E.** As C but for rs1006353. **F.** As D but for rs1006353. **G.** As C but for rs11135484. **H.** As D but for rs11135484.

Having identified a set of SNPs overlapping T-bet binding sites, we next asked whether these sequence variants altered T-bet binding. The traditional pull-down technique is time intensive and semi-quantitative. Therefore, we explored whether transcription factor binding could be assayed using a flow cytometric readout. In this technique, which we call OligoFlow, a fluorochrome-labelled antibody for the transcription factor of interest is added to the oligonucleotide-bead / lysate mix, and the Median Fluorescence Intensity (MFI) of the beads is assessed by flow cytometry as a quantitative measure of binding efficiency (Fig 3A).

To validate this new technique, a positive control oligonucleotide (Motif+) was designed to incorporate the previously identified consensus sequence [54] surrounded by non-specific sequence (S3 Table). A negative control oligo (Motif-) incorporated mutations of two key residues within the motif. OligoFlow was conducted with lysate from either the YT human cell line, which constitutively expresses T-bet [59], or lysate from primary human CD4^+^ cells polarised under Th1 conditions in culture. The positive and negative control oligonucleotides showed a clear difference in MFI (Fig 3A) and thus OligoFlow can successfully discriminate positive and negative transcription factor binding events.

We then proceeded to test a subset of our T-bet hit-SNPs that were also associated with H3K27ac or near genes of immunological interest. SNPs that showed differential binding were tested at least five times. Within each experiment, the MFI of each allele was normalised to the MFI of the negative control and significantly altered binding between alleles across all experiments was assessed using a paired t-test. Three T-bet hit-SNPs exhibited significantly different binding to the two alleles; rs1465321, located within the second intron of *IL18R1*, rs1006353, 22.5 kb upstream of *MTIF3*, and rs11135484, within an intron of *ERAP2* (Fig 3B). Differential T-bet binding to the two alleles of rs1465321 were confirmed by traditional oligonucleotide pull-down (S2 Fig). All 3 SNPs are [A/G] with A as the minor allele. In each case, allele A is also in LD with alleles associated with for the trait under investigation. rs1465321 is in high LD with multiple SNPs associated with celiac disease, including rs13015714 and rs917997, identified as the strongest risk alleles for celiac disease in 2q12.1 [18,60], with the disease-associated alleles linked to reduced *IL18RAP* expression [60]. rs1465321 and rs11135484 have also been associated with Crohn’s disease [18,22,60,61], but not in a more recent study [21]. For rs1465321 and rs1006353, the minor disease-associated A allele binds T-bet less strongly than the G allele (Fig 3C–3F). In contrast, for rs11135484, the A allele binds T-bet more strongly than the G allele (Fig 3G and 3H). We conclude that disease-associated genetic variants can alter T-bet binding to DNA *in vitro*.

### SNPs affecting T-bet binding cannot reliably be identified by motif analysis

**Fig 4:**
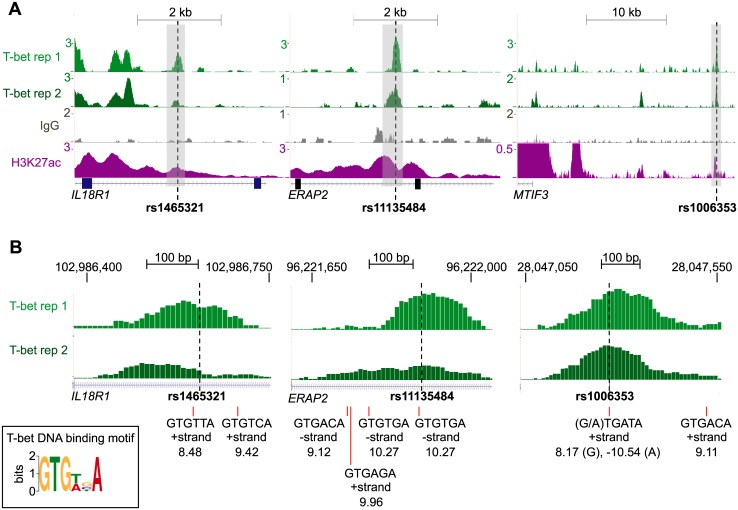
Motif analysis does not reliably predict impact on T-bet binding. **A.** T-bet binding, IgG control and H3K27ac modification (ChIP-seq reads/million) at the genomic regions surrounding the SNPs rs1465321 (left), rs11135484 (center) and rs1006353 (right). The location of the SNPs are indicated by dashed vertical lines. The regions highlighted in grey are expanded in B. **B.** Expanded view of T-bet binding at the regions highlighted in grey in A. The locations of sequences matching the identified T-bet DNA binding motif (inset) are marked by red lines, together with their score (a negative value indicates a poor match). Only rs1006353 overlaps a T-bet DNA binding motif and the A allele is predicted to disrupt the motif and T-bet binding.

Motif analysis has often been used to predict transcription factor binding sites affected by genetic variants. We previously derived a consensus T-bet motif from T-bet binding sites in human Th1 cells [54] and repeated this analysis with duplicate T-bet ChIP-seq data (Fig 4). The three T-bet hit-SNPs that showed altered binding in OligoFlow were then examined for whether they disrupted such a T-bet binding motif. In the case of rs1006353, the G allele formed part of a T-bet binding motif, whereas the A allele abolished this binding site (Fig 4B). However, neither of the other two SNPs, rs1465321 and rs11135484, overlapped a predicted T-bet binding motif (Fig 4B). Thus, over-reliance on motif analysis can result in SNPs with the potential to alter transcription factor binding sites being missed and highlights the importance of using experimental validation to confirm binding of the relevant transcription factor.

### Differential T-bet binding at disease variants *in vivo*

**Fig 5:**
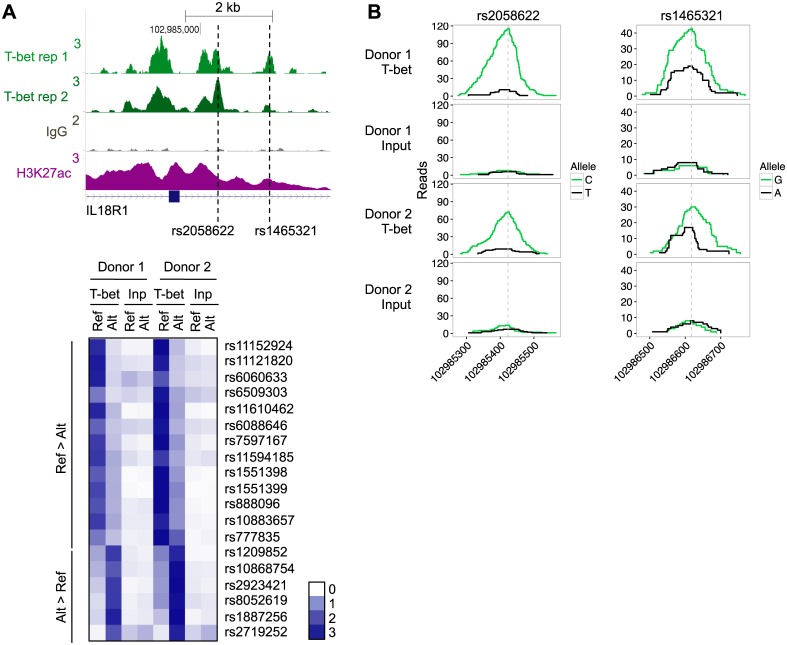
Genetic variants alter T-bet binding *in vivo*. **A.** Genomic context of rs1465321 and rs2058622, which is in high LD (r^2^ = 1.0) with rs1465321. **B.** T-bet ChIP and input sequencing reads that cross rs2058622 (chr2: 102985274–102985565; left) or rs1465321 (chr2: 102986477–102986768; right) in two donors heterozygous for rs1465321. In each case, the number of reads that match the reference allele are shown in black and the alternative allele in green. **C.** T-bet ChIP and input (Inp) sequencing reads at the set of 19 additional heterozygous SNPs that exhibited allelic imbalanced T-bet binding. For each SNP, the color shows fold-enrichment in the number of sequencing reads matching the Ref or Alt allele, relative to the average number of reads across all samples, as indicated by the scale on the right hand side. SNPs are divided into those exhibiting greater T-bet binding to the reference (Ref) allele (Ref > Alt, top) or the alternative (Alt) allele (Alt > Ref, bottom).

We next sought to confirm that T-bet exhibited differential binding to disease-associated SNPs *in vivo*. We focused on rs1465321, because it lies within the *IL18R1/IL18RAP* gene locus that we have previously identified as a T-bet target [54] and because disease-associated alleles in high LD are associated with reduced *IL18RAP* expression and disease [60]. Primary naive CD4+ T cells were purified from the peripheral blood of two individuals heterozygous for this SNP and were polarised into the Th1 lineage. We then performed ChIP-seq for T-bet in these cells, as previously described [54]. We aligned the reads for the T-bet ChIP-enriched DNA and input controls to the reference human genome and then counted the number of reads matching the major or minor alleles in the inputs and ChIP samples. In the input DNA samples, there were approximately equal numbers of reads for the two alleles in both individuals. In comparison, the T-bet ChIP reads showed significantly lower enrichment for the minor A allele in both donors (Fig 5A and 5B). There was also a significant allelic imbalance for T-bet binding at the neighbouring SNP rs2058622, which is in high LD (r^2^ = 1.0) with rs1465321 (Fig 5A and 5B). To determine whether T-bet exhibited allelic imbalanced binding at any other loci, we identified all SNPs that exhibited heterozygosity in both individuals. Of the heterozygous SNPs that overlapped a T-bet binding site, 19 exhibited significant allelic imbalanced binding in both donors after adjustment for multiple hypothesis testing (Fig 5C, S4 Table). These included the IBD-associated SNPs rs1551398 and rs1551399 [21], situated 86bp apart and downstream of *TRIB1* (Fig 5C, S1 and S3 Figs). We conclude that the two alleles of rs1465321 exhibit different levels of T-bet binding *in vivo*, with the disease associated A allele bound significantly less, and that the credible IBD variants rs1551398 and rs1551399 also influence T-bet binding.

### Regulation of *Il18rap and Trib1* expression in T-bet^-/-^ Th1 cells

**Fig 6:**
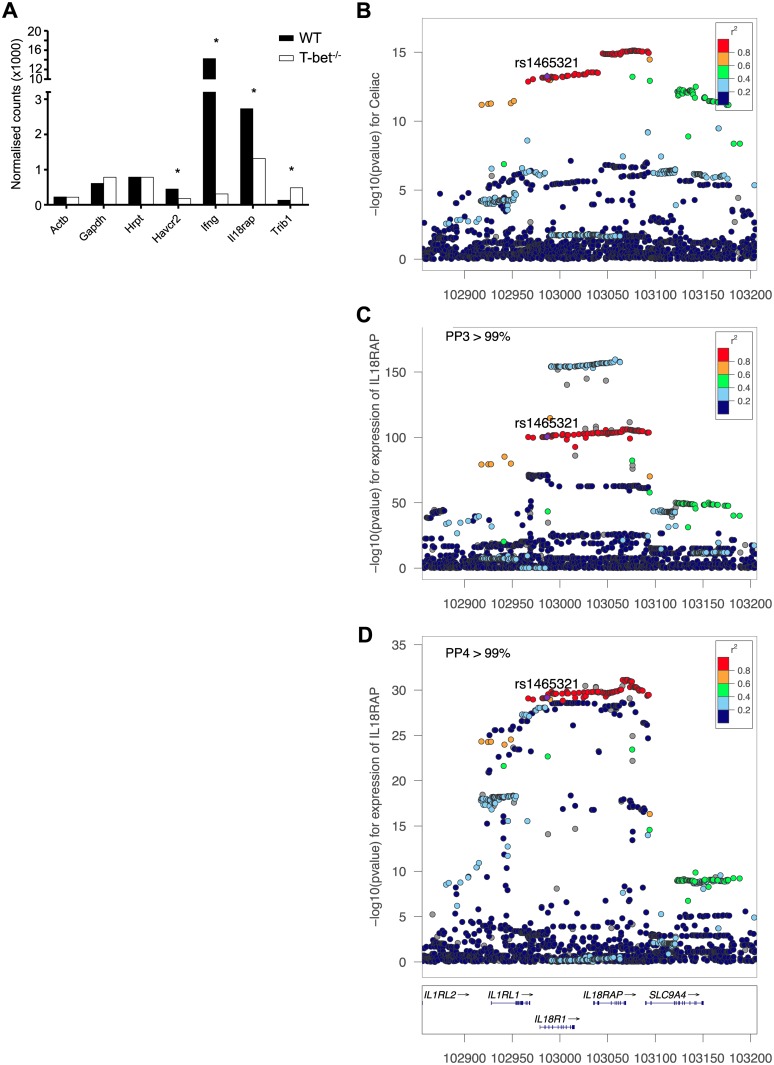
rs1465321 is an eQTL for *IL18RAP* and celiac disease. **A**. RNA abundance (size factor-normalised counts) for selected genes in wild type (WT) and T-bet deficient (T-bet KO) naive lymphocytes cultured under Th1 polarising conditions. * significant change in expression (p<0.05, Wald test after Benjamini-Hochberg correction). **B.** “Locus-zoom” plot showing the distribution of association p-values for celiac disease in the *IL18RAP/IL18R1* chromosome region (genes shown below panel D). The x-axis shows the chromosome position. Colors show the level of linkage disequilibrium with rs1465321, which is indicated with a purple spot. **C.** “Locus-zoom” plot showing the distribution of association p-values for *IL18RAP* eQTL in 1,214 whole blood RNA samples. The Bayesian statistic for colocalisation with the celiac disease signal shows a posterior probability *against* colocalisation (PP3) greater than 99%, indicating that this primary whole blood *IL18RAP* eQTL signal is not compatible with a shared causal variant with celiac disease. **D.** “Locus-zoom” plot showing the secondary *IL18RAP* eQTL (conditional on rs1985329) signal in the same 1,214 whole blood RNA samples. For this secondary signal, the Bayesian statistic for colocalisation with the celiac disease signal shows a posterior probability *in favour of* colocalisation (PP4) greater than 99%, indicating that this secondary whole blood *IL18RAP* eQTL signal is compatible with a shared causal variant with celiac disease.

Having identified rs1465321, rs2058622, rs1551398 and rs1551399 as disease associated SNPs that modulate T-bet binding *in vivo*, we next determined whether there was a functional relationship between T-bet binding and the genes associated with these SNPs. rs1465321 and rs2058622 are in high LD with SNPs associated with low expression of *IL18RAP* in celiac disease [60]. The IBD-associated SNPs rs15513998 and rs1551399 are associated with *TRIB1* [21]. To determine whether there was a functional relationship between T-bet binding and *IL18RAP* and *TRIB1* expression, we compared gene expression profiles of wild type and T-bet^-/-^ naïve CD4+ T cells polarised in Th1 conditions. As expected, genes known to be positively regulated by T-bet were significantly downregulated in T-bet^-/-^ cells, including Interferon-γ (*Ifng*) and *Tim-3* (*Havcr2*), while the housekeeping genes *Gapdh*, *Actb* and *Hprt* remained unchanged (Fig 6A). *Il18rap* was also significantly downregulated in the absence of T-bet, implying a positive regulatory role for T-bet in modulating its expression (Fig 6A). In contrast, Trib1 was significantly upregulated in T-bet^-/-^ cells, implying that T-bet functions to repress this gene. Consistent with a direct role for T-bet in regulating *Il18rap and Trib1* expression, multiple T-bet binding sites were located within intronic regions of murine *Il18rap* and downstream of *Trib1* (S4 Fig). Thus, these data support a direct role for T-bet binding in the regulation of *IL18RAP* and *TRIB1* expression.

### rs1465321 is associated with *IL18RAP* expression and co-localises with celiac disease risk

We next explored whether the genotype of rs1465321 could control the expression of nearby genes and how this potential eQTL related to celiac disease susceptibility (Fig 6B). Celiac disease association was based on a case control association study of 12,041 celiac disease cases and 12,228 controls [23]. Using a gene expression dataset of 1,214 samples [62] we found a strong correlation between rs1465321 genotypes and *IL18RAP* expression level (p<10^−100^, Fig 6C). No other gene showed a significant association with rs1465321. However, this SNP did not display the greatest eQTL association compared with other variants in the region, which could suggest a lack of a causal role. Moreover, using a previously developed methodology [63], we established that the eQTL and disease association signals in the *IL18RAP* regions were unlikely to be driven by the same genetic variant (posterior probability supporting a shared variant < 1%, Fig 6C). However, a stepwise regression analysis of the eQTL data shows that after accounting for the primary eQTL signal (conditional on rs1985329), a second eQTL association was clearly detectable (p<10^−30^). This suggested that at least two independent variants, with distinct biological mechanisms, are controlling *IL18RAP* mRNA expression. Interestingly, this secondary eQTL signal co-localized with the celiac disease risk signal (Fig 6D, posterior probability supporting a shared variant > 99%). Moreover, rs1465321 is one of the most strongly associated genetic variants for this secondary eQTL signal, with the disease-associated A allele, which exhibited reduced T-bet binding, associated with reduced *IL18RAP* expression. Therefore, our combined fine-mapping disease eQTL data are consistent with rs1465321 affecting *IL18RAP* expression through altered binding of T-bet.

## Discussion

We have found that IBD and celiac disease-associated SNPs are significantly enriched at T-bet binding sites. Surprisingly, this association is not observed for RA or psoriasis, suggesting it may be specific for mucosal inflammatory disease. Furthermore, we have identified genetic variants that alter T-bet binding to DNA, both *in vitro* and *in vivo*, including rs1465321, which we also identify as an eQTL for *IL18RAP* and celiac disease. Thus, these data provide a mechanistic explanation for why a single base change at this locus is associated with changes in gene expression and disease risk.

Although some studies have identified sequence variants that modulate transcription factor binding, alterations in the binding of Th lineage-specifying factors at disease-associated variants has not previously been identified. Our discovery that SNPs associated with IBD and celiac disease alter T-bet occupancy reveals that genetic variants can have a significant impact on the function of key master regulator transcription factors that govern cell fate. The strong association of T-bet binding sites with mucosal autoimmune/inflammatory diseases suggests that other disease-associated variants also act to alter the binding of this critical immune regulator, with important consequences for T cell polarisation and lineage-specific gene expression.

That T-bet binding sites are associated with mucosal autoimmune disease, but not with RA or psoriasis is somewhat surprising, because all of these diseases have been linked to aberrant Th1 responses [2]. However, mucosal disease is more strongly associated with aberrant Th17 responses, which are repressed by T-bet [3,4,64,65], providing a mechanistic rationale for our findings. We and others have recently shown that T-bet plays a critical and non-redundant role in the function of ILCs [2,7,11–15]. It is therefore feasible that the association of mucosal autoimmune disease-associated SNPs with T-bet binding sites reflects alterations to T-bet binding in ILCs, which have a key regulatory role at mucosal surfaces. Expanding our fGWAS analysis to other autoimmune conditions will be necessary to fully establish the specificity of the association of T-bet with SNPs associated with mucosal disease.

Significantly, we have demonstrated that T-bet binding is enriched at disease-associated SNPs that have high posterior probabilities [21]. This suggests that more T-bet bound variants will be discovered when other IBD loci are subjected to fine-mapping analysis. We further found that the disease-associated alleles of rs1551398 and rs1551399 both reduce T-bet binding *in vivo*. These SNPs are located upstream of *TRIB1*, a gene that is upregulated in the mucosa of both UC and CD patients [66]. Consistent with this, we find that T-bet functions to repress *Trib1* expression, suggesting that the disease-associated alleles may increase disease risk by abrogating T-bet-mediated repression of this gene. T-bet also binds at 2 other sites near TRIB1 (rs28510097 and rs1551400) and, together, these 4 SNPs account for 55% posterior probability of association for this locus [21].

We also identified rs1465321, located within an intron of *IL18R1*, to exhibit allele-imbalanced T-bet binding. This SNP is an eQTL for *IL18RAP* and celiac disease risk, with the minor disease-associated allele linked with reduced T-bet binding and *IL18RAP* gene expression. IL18RAP and IL18R1 together form the IL-18 receptor. Signaling through this receptor, IL-18 synergizes with IL-12 to induce IFNγ. rs1465321 is in high LD with the lead SNP in this locus for celiac disease [60]. Although our data are consistent with rs1465321 altering *IL18RAP* expression through altered binding of T-bet, we cannot rule out that variants in strong LD with rs1465321 could also be causal, such as rs2058622 that also exhibits allele-imbalanced T-bet binding. Given that T-bet acts through multiple sites to regulate its target genes [52, 54, 67, 68], it is likely to be the combined effect of the haplotype that is relevant. ChIP-seq for T-bet in individuals heterozygous for other disease-associated SNPs will likely reveal further examples of genetic variants that modulate T-bet binding.

Our finding that there are two independent eQTLs for *IL18RAP*, and that only one of these is associated with celiac disease (Fig 6), suggests that the level of *IL18RAP* expression may not be functionally relevant for disease susceptibility. Alternatively, it is possible that the two independent eQTLs for *IL18RAP* represent different enhancers that mediate transcriptional activation in different cells or in response to different stimuli, and that *IL18RAP* expression level is only relevant for celiac disease in one cell type or in response to a particular signal.

Attempts to determine the likely effect of non-coding sequence variants have mostly focused on identifying overlapping transcription factor binding motifs or overlapping sites of transcription factor binding, DNase I hypersensitivity or DNA and histone modification. Our analysis of allele-specific T-bet ChIP-seq data shows that genetic variants within transcription factor binding sites do not necessarily alter transcription factor binding. Similarly, genetic variants that do impact transcription factor binding do not necessarily lie within the predicted motif. Thus, confirmation of allele-specific binding events is necessary to confirm that a SNP does indeed impact transcription factor function and provides a mechanistic link between genetic variation and disease risk.

We have established the feasibility of using flow cytometry to assay allelic effects on transcription factor binding, and validated this technique through both the traditional pull-down assay and allele-specific ChIP-seq. As flow cytometric methods can be easily automated, this method provides a more rapid means to assay large numbers of allelic variants compared to traditional pull-down methods. Using this OligoFlow method, we identified alterations in T-bet binding at rs11135484, in high LD with a SNP associated with Crohn’s disease and with rs1006353, the closest neighbor of which is *MTIF3*, associated with body mass index [69]. Interestingly, T-bet has been linked with regulation of insulin sensitivity and visceral adiposity [70].

In summary, we have identified a specific association between T-bet binding sites and mucosal autoimmune disease variants and determined that such genetic variants modulate T-bet binding in cells. This suggests that altered binding of T cell master regulators can predispose individuals to specific autoimmune and inflammatory conditions. This study establishes a scalable method that can be used to explore the impact of genetic variation on the function of other lineage-specifying transcriptional factors. These insights will identify molecular mechanisms that underlie the genetic basis of autoimmune diseases and suggest new therapies for their treatment.

## Materials and methods

### T-bet hit-SNP identification

ChIP-seq for T-bet in human Th1 cells was performed previously [53–54] (GEO accessions: GSE31320 and GSE62486) and binding sites were identified from the merged dataset with MACS 1.4 (p<10^−7^) [71]. The positions of T-bet peaks were identified relative to gene transcription start sites annotated in RefSeq. The GWAS catalogue was downloaded from the NHGRI [55] on December 12th 2014. SNPs were checked against dbSNP and 4 SNPs called ‘suspect’ removed. SNPs that had been merged with other IDs were checked against HapMap3 and the ID given in HapMap3 used in downstream analysis. SNPs not in HapMap3 were removed, giving 13,936 autosomal SNPs in the final analysis. Data were analysed using the bioconductor snpMatrix programme (recently updated to snpStats) [72, 73]. SNPs in high LD (r^2^> 0.8 with a SNP from the GWAS catalogue) were obtained from HapMap3 [74], giving a total of 127,594 SNPs. These were then overlapped with the T-bet binding sites. To identify the number of independent LD blocks were represented by the 926 T-bet bound SNPs, we used the *SNPclip* module of LDlink to reduce any SNPs in high LD to a single tag SNP, using a R^2^ threshold of 0.8 and a MAF (Minimum Allele Frequency) threshold of 0.01.

### Comparison to H3K27ac and DHS

ChIP-seq data for IgG, H3K27ac and total H3 were taken from GSE62486. Sequence reads were trimmed to remove low quality bases and to remove adapters and aligned using Bowtie (default settings) to hg19. Peaks of H3K27ac were identified with MACS (p<10^−7^) [71]. DHS data were obtained from ENCODE (GEO accession GSM736592) [75,76]. Average binding profiles were calculated across 4 kb windows centred on hit-SNPs using ngsplot [77]. Data were visualized on the UCSC genome browser by calculating tag density in 10bp windows, normalizing to reads per million total reads and subtracting background (input for T-bet and H3 for H3K27ac), as described [54].

### Human SNP genotyping

Individuals heterozygous for rs1465321 were identified from the Twins UK cohort at the Guy’s and St Thomas’ NHS Foundation Trust (GSTT) Bioresource, where HumanHap610Q Illumina array data is available for all registered participants. The Illlumina calling algorithm [78] was used to assign genotypes from array data. Before imputation, quality controls were applied, with exclusion of all samples with: (1) call rate <98%, (2) heterozygosity across all SNPs ≥2 standard deviations from the sample mean; (3) evidence of non-European ancestry as assessed by PCA comparison with HapMap3 populations; (4) observed pairwise IBD probabilities suggestive of sample identity errors. We also corrected zygosity based on IBD probabilities. Quality controls were also applied to each individual SNP using the following exclusion criteria: (1) Hardy-Weinberg p-value <10^−6^ (assessed in a set of unrelated samples); (2) MAF <1% (assessed in a set of unrelated samples); (3) SNP call rate <97% (SNPs with MAF ≥5%) or < 99% (for 1% ≤ MAF < 5%). Finally all the alleles were aligned to the forward strand of HapMap2. After completion of both sample and SNP quality controls checks, imputation was performed using the IMPUTE software package (v2) [79] using HapMap2 as a reference panel (HapMap2, rel. 22, combined CEU+YRI+ASN panels). Heterozygous SNPs were selected using PLINK (version 1.0.7) [80] “—recode-rlist” option on the imputed dataset. A final QC stage was applied on all the heterozygous SNPs, excluding all those polymorphisms with an imputation quality score ≤ 0.8.

### Allele-specific T-bet ChIP-seq

In accordance with the Department of Health’s Research Governance Framework for Health and Social Care, ethical approval for this study was gained from the South London Research Ethics Committee (Ref:15/LO/0151), and from the Department of Research and Development at GSTT NHS Trust (Ref:RJ115/N122). Approval was also gained from the GSTT National Institute of Health Research (NIHR) Bioresource for recruitment of individuals registered on the Bioresource and heterozygous for rs1465321. All of the subjects in this study gave written consent. Blood was taken from two individuals heterozygous for the desired SNP. CD4+ T cells were purified from whole blood leukocytes using CD4 microbeads (Miltenyi Biotec) and naïve CD4+ T-cells sorted by FACS selection for CD4+ CD45RA+ CD4RO- CD25- CCR7+ cells. Sorted naïve T-cells were activated with anti-CD3/CD28 and polarized under Th1 conditions (IL2, IL12 and anti-IL4) for 7 days [54]. Cells were then crosslinked and ChIP-seq for T-bet performed with a custom-made polyclonal antibody [54]. Libraries were quantified using the KAPA library quantification kit and sequenced (150 bp single-end) with an Illumina NextSeq. Sequence reads were trimmed and aligned to hg19 as before. Peak regions for both donor 1 and 2 were identified separately using MACS 1.4. Broad shallow peaks were filtered, intersecting peaks identified with Bedtools (n = 8185), and then narrowed to the central 400 bp. Potential SNP variants within these intersecting peak regions were extracted from dbSNP version 138 (assembly hg19, n = 490,310). SNP sites for further analysis were determined from the Bowtie aligned bam files as containing >1 reads with both Ref and Alt bases in both ChIP and Input samples from both donors (n = 9058). This list was then compared to the set of heterozygous SNPs identified by the SNP array analysis (n = 2621 high confidence heterozygous SNPs). Reads surrounding these sites were extracted into R using the Bioconductor Rsamtools and GenomicRanges packages. The reads were split by Ref and Alt alignment for visualization using the GenomicAlignments package.

To test whether T-bet exhibited allelic imbalanced binding at rs1465321 and at SNPs in high LD, we used a binomial test. Donor 1 and 2 p-values were combined using the Fisher method. rs1465321 and rs2058622 showed significant allelic imbalance (p<0.01) in the T-bet ChIP samples and allelic balance (p>0.1) in the Input samples. To identify other heterozygous SNPs that exhibited allelic-imbalanced T-bet binding, we used a binomial test to identify heterozygous SNPs at which significantly more reads were reported for one allele compared to the other in both T-bet ChIP samples (Benjamini-Hochberg adjusted p<0.05) but not imbalanced in the Input samples from either donor (unadjusted p>0.4). This produced a list of 19 additional SNPs (S4 Table).

T-bet ChIP-seq data from donors 1 and 2 heterozygous for rs1465321 are available at GEO under accession GSE81881.

### RNA-seq analysis

Data-sets for wild-type and T-bet deficient CD4+ T cells polarised in Th1 and Th2 conditions were obtained from GEO (GSE38808). Raw reads were aligned to the mm10 build of the murine genome using Subread [81], and subsequently mapped to RefSeq genes using featureCounts [82]. DESeq2 was used to normalise read counts by size factors, and call differentially regulated genes using an empirical Bayes model and the Wald test followed by Benjamini-Hochberg correction for multiple testing [83].

### Motif analysis

The presence of T-bet motifs was assessed using FIMO [84] using previously compiled matrices for T-bet binding obtained by ChIP-seq [54]. Sequences for T-bet binding sites were obtained from the hg19 reference genome and SNPs were manually altered to the alternative allele.

### fGWAS

*fGWAS* analysis was performed as described in [56] using *fGWAS* version 0.3.3 with case control setting. Data were prepared for *fGWAS* using R and the *GenomicRanges* package to compute overlap between binding sites and SNPs. Publicly available GWAS data were downloaded from the websites of the relevant consortiums for UC, Crohn’s disease (http://www.ibdgenetics.org/downloads.html), coronary artery disease (http://www.cardiogramplusc4d.org/downloads/), and rheumatoid arthritis (http://plaza.umin.ac.jp/~yokada/datasource/software.htm). Psoriasis data are from [85]. T-bet binding sites were identified as described above. ENCODE ChIP-seq, FAIRE-seq and DNaseI hypersensitivity datasets were obtained from the ENCODE website in bed format (http://ftp.ebi.ac.uk/pub/databases/ensembl/encode/integration_data_jan2011). The complete ENCODE datasets combines DNaseI (125 annotations), FAIRE-seq (24 annotations), histone marks (117 annotations) and transcription factor binding site datasets (S2 Table). In addition, we included GATA3 binding sites in Th1 and Th2 cells (GSE31320) [54], FOXP3 binding sites in Tregs [86], NF-κB binding sites in lymphoblastoid cells [47] (GSE19486), and H3K27ac [58] and DHS [57] in Th1 and Th2 cells.

### eQTL analysis

Celiac disease association was based on a case control association study of 12,041 celiac disease cases and 12,228 controls [23]. Gene expression data were taken from [62]. eQTL analysis was performed as described [63]. eQTL p-values were obtained by fitting a linear trend test regression between the expression of each gene and all variants 200 kb upstream and downstream from each probe. Posterior computation was performed as described [63]. The regional association plots for the eQTL and Biomarker datasets were created using LocusZoom [87] (http://csg.sph.umich.edu/locuszoom/).

Colocalisation analysis was performed using the R package COLOC [63] based on single variant summary statistics (log odds ratio, standard error for the log odds ratio for case control and effect size and standard error for effect size for eQTL study, in addition to MAF and physical position for each variant) and with the default settings provided with the R package.

### Cell culture for oligonucleotide pull-down assays

Human CD4^+^ cells were isolated from buffy coats (UK National Blood Service, used under REC reference number 10/H0804/65 from SE London Research Ethics Committee 2) using RosetteSep human CD4+ T cell enrichment cocktail (STEMCELL Technologies) according to manufacturer’s instructions and polarised towards a Th1 phenotype in supplemented RPMI as described in above. Cells were harvested after a total of seven days of culture. YT cells were cultured in RPMI medium (PAA) supplemented with 50 units/ml penicillin, 50 μg/ml streptomycin (Gibco), 10 mM HEPES buffer solution (Fisher Scientific), 1 mM sodium pyruvate (Gibco), 1 × minimum essential medium-non essential amino acids (Gibco), 2 mM L-glutamine (Gibco) and 10% foetal bovine serum (PAA). All cells were maintained at 37°C in 5% CO2.

### OligoFlow and pull-downs

Forward and reverse single-stranded oligos (Integrated DNA Technologies, S3 Table) for each allele of each SNP were annealed by incubating at 94°C for 5 mins, 65°C for 10 mins, 25°C for 10 mins and 4°C thereafter in annealing buffer (50 mM Tris pH 8, 7 mM MgCl_2_ and 1 mM DTT). For pull-down and western blot, 20μl of streptavidin agarose beads (Sigma) were used per sample. For OligoFlow, 50 μl of Sphero streptavidin polystyrene particles (Spherotech #SVP-100-4) were used per sample. Beads were washed twice in PBS and then once in annealing buffer. Beads were then incubated with double-stranded oligonucleotides for 1 hr at 4°C, washed twice in oligo buffer (10 mM Tris pH 8, 100 mM NaCl, 0.1 mM EDTA, 1 mM DTT, 5% glycerol, 1 mg/ml BSA Fraction V, 20 μg/ml dI/dC (Sigma, P4929) and Complete protease inhibitor (Roche) and finally resuspended in 450 μl oligo buffer. Cells (30 million per sample) were washed twice in PBS and lysed in 1 ml hypotonic buffer (20 mM HEPES pH 8, 10 mM KCl, 1 mM MgCl2, 0.1% Triton X-100, 5% glycerol, 1 mM DTT and Complete protease inhibitor) on ice for 5 mins. Lysed cells were pelleted and resuspended in 150 μl hypertonic buffer (20 mM HEPES pH 8, 400 mM NaCl, 1 mM EDTA, 0.1% Triton X-100, 5% glycerol, 1 mM DTT and Complete protease inhibitor). Debris was pelleted, 180 μl of supernatant containing nuclear extract added to the beads and incubated on a rotor for 1 hour at 4°C. For western blotting, samples were then washed three times in oligo buffer and resuspended in SDS loading buffer. For OligoFlow, 0.25 μg of anti-T-bet Alexa647 antibody (clone 4B10, BioLegend) was added and samples incubated for a further 1 hr at 4°C. Data (at least 30,000 events) were acquired on a FACSCanto flow cytometer (BD Biosciences).

### Western blotting

Oligonucleotide pull-down samples were heated in SDS loading buffer before transfer to nitrocellulose membrane. Samples were blocked in 5% milk in TBS-T (1 hr, RT) and incubated with 1:1000 anti-T-bet (clone eBio4B10 (eBioscience); 4°C overnight). Blots were washed before addition of anti-mouse-HRP (GE Healthcare) and visualised with Enhanced Chemiluminescent Substrate (PerkinElmer) and exposed to film.

## Supporting information

S1 Fig T-bet binding at disease-associated SNPs. **A.** T-bet binding at further example T-bet hit-SNPs. The number of sequencing reads from T-bet, IgG control and H3K27ac ChIP-enriched DNA are plotted per million input-subtracted total reads and aligned with the human genome. DNaseI hypersensitivity data (2 replicates) are from ENCODE. **B.** The frequency distribution of posterior probabilities for association with IBD (from [21]) for SNPs [21] that do not overlap (left) or that do overlap (right) a T-bet binding site. SNPs that overlap a T-bet binding site tend to have a higher posterior probability (binomial regression, p = 6.3x10^-6^).
(PDF)

S2 Fig Confirmation of altered T-bet binding at rs1465321 by oligonucleotide pull-down. **A** The effect of the different rs1465321 alleles on T-bet binding was assessed by oligonucleotide pull-down followed by immunoblotting—a representative blot for rs1465321 is shown. **B** Quantification of immunoblot band density, normalised to allele A. Error bars show standard deviation (n = 4). The difference in T-bet binding to the G compared to A allele of rs1465321 was significant (p = 0.048, paired t-test).
(PDF)

S3 Fig Heterozygous SNPs showing imbalanced T-bet binding. T-bet ChIP and input sequencing reads that cross rs888096, rs1551399 and rs1551398 in two donors. In each case, the number of reads that match the reference allele are shown in black and the alternative allele in green.
(PDF)

S4 Fig T-bet binding at the mouse *Il18r1/Il18rap* and *Trib1* loci. The number of sequencing reads from T-bet ChIP-enriched DNA from WT (GSM998272 and GSM836124) and T-bet KO mouse Th1 cells (GSM998273) plotted per million input-subtracted total reads and aligned with the mouse genome (mm9) at the *Il18r1/Il18rap* (A) and the *Trib1* (B) loci.
(PDF)

S1 Table Summary data for T-bet hit-SNPs. (XLSX)

S2 Table Functional annotation datasets used for fGWAS. (XLS)

S3 Table Oligonucleotide sequences used for OligoFlow. (DOCX)

S4 Table Heterozygous SNPs showing imbalanced T-bet binding *in vivo*. (XLSX)
