# Supplementary figures and images for: Genetic variants alter T-bet binding and gene expression in mucosal inflammatory disease
Source: PLoS Genet. 2017 Feb 10;13(2):e1006587. doi: 10.1371/journal.pgen.1006587 (PMC5328407; doi:10.1371/journal.pgen.1006587)

S1 Figure

A

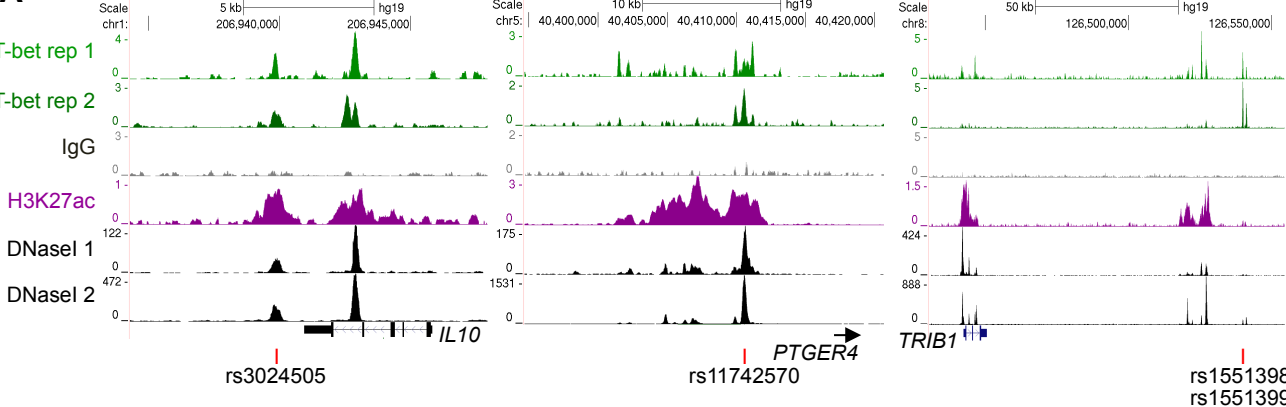

B

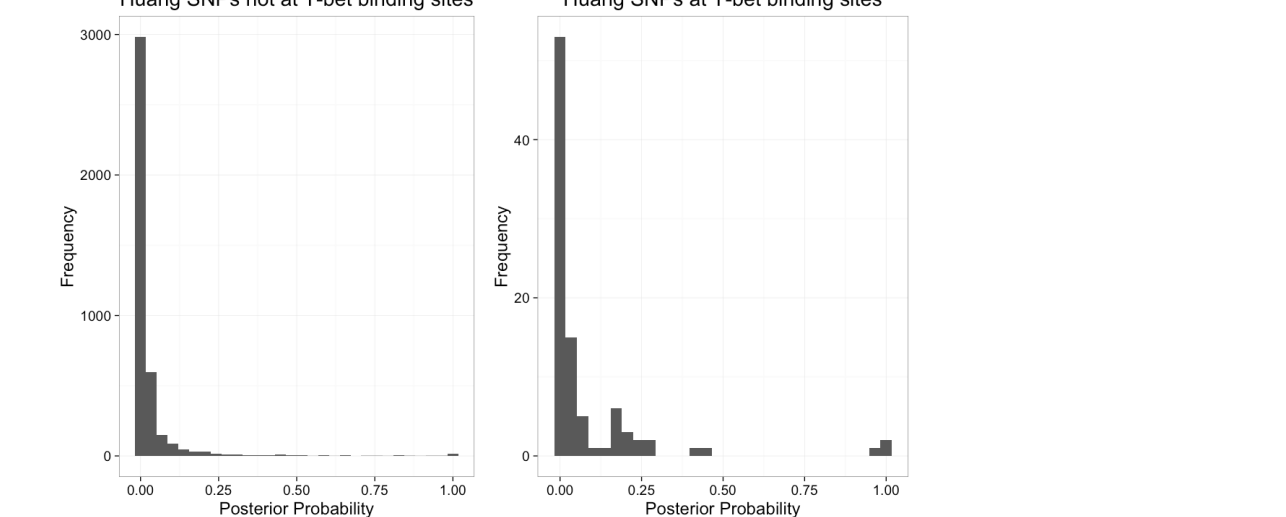

Supplement: S1 Fig — A. T-bet binding at further example T-bet hit-SNPs. The number of sequencing reads from T-bet, IgG control and H3K27ac ChIP-enriched DNA are plotted per million input-subtracted total reads and aligned with the human genome. DNaseI hypersensitivity data (2 replicates) are from ENCODE. B. The frequency distribution of posterior probabilities for association with IBD (from [21]) for SNPs [21] that do not overlap (left) or that do overlap (right) a T-bet binding site. SNPs that overlap a T-bet binding site tend to have a higher posterior probability (binomial regression, p = 6.3x10^-6^). (PDF) [file pgen.1006587.s001.pdf]

S2 Figure

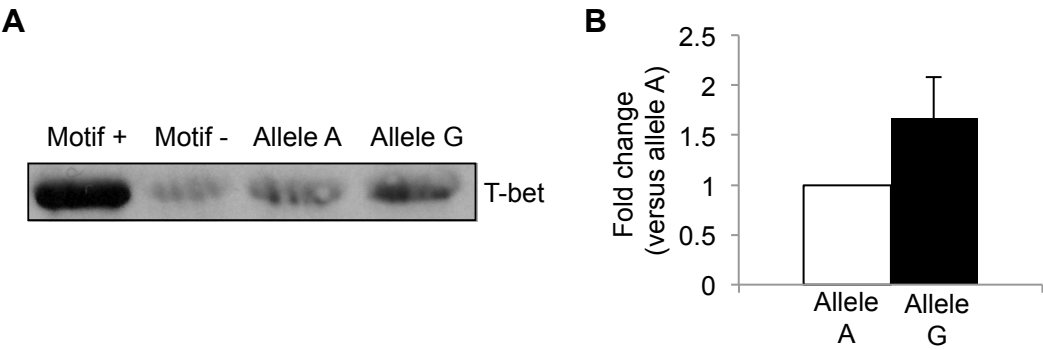

Supplement: S2 Fig — A The effect of the different rs1465321 alleles on T-bet binding was assessed by oligonucleotide pull-down followed by immunoblotting—a representative blot for rs1465321 is shown. B Quantification of immunoblot band density, normalised to allele A. Error bars show standard deviation (n = 4). The difference in T-bet binding to the G compared to A allele of rs1465321 was significant (p = 0.048, paired t-test). (PDF) [file pgen.1006587.s002.pdf]

S3 Figure

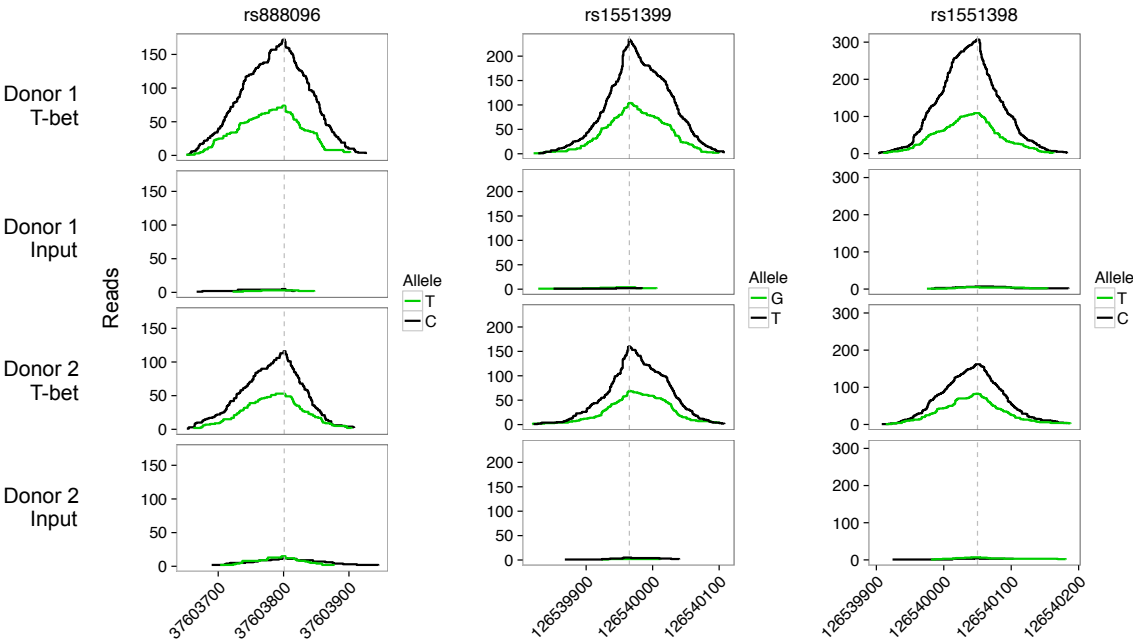

Supplement: S3 Fig — T-bet ChIP and input sequencing reads that cross rs888096, rs1551399 and rs1551398 in two donors. In each case, the number of reads that match the reference allele are shown in black and the alternative allele in green. (PDF) [file pgen.1006587.s003.pdf]

S4 Figure

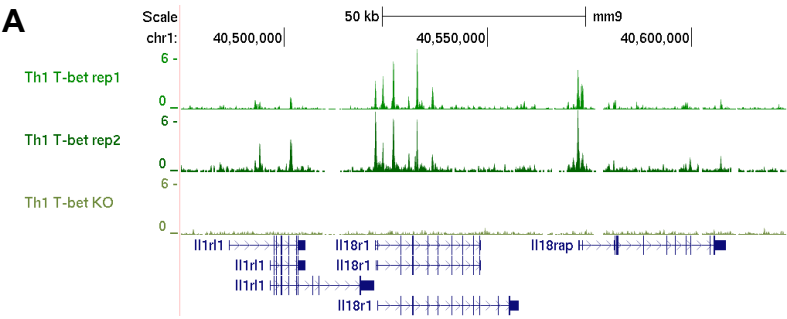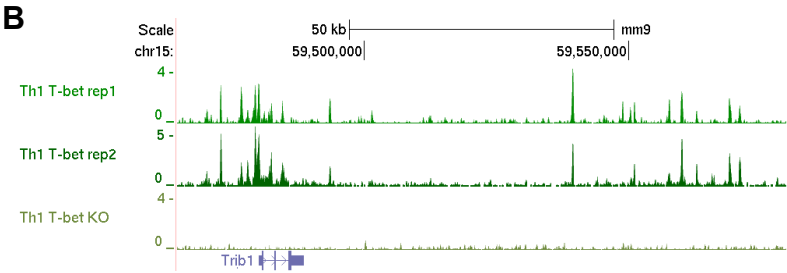

Supplement: S4 Fig — The number of sequencing reads from T-bet ChIP-enriched DNA from WT (GSM998272 and GSM836124) and T-bet KO mouse Th1 cells (GSM998273) plotted per million input-subtracted total reads and aligned with the mouse genome (mm9) at the Il18r1/Il18rap (A) and the Trib1 (B) loci. (PDF) [file pgen.1006587.s004.pdf]
