# Supplementary material for: Genetic variants alter T-bet binding and gene expression in mucosal inflammatory disease
Source: PLoS Genet. 2017 Feb 10;13(2):e1006587. doi: 10.1371/journal.pgen.1006587 (PMC5328407; doi:10.1371/journal.pgen.1006587)
Supplement: S3 Table — (DOCX) [file pgen.1006587.s007.docx]

**S3 Table. Sequences of oligonucleotides used in OligoFlow**

| **Name** | **Sequence** |
| --- | --- |
| T-bet Positive Forward | Biotin-TTAGCTAGTCGGCGCTATAGTTTTCACACCTGATCGTAGATCGCTAGCTAGTA |
| T-bet Positive Reverse | TACTAGCTAGCGATCTACGATCAGGTGTGAAAACTATAGCGCCGACTAGCTAA |
| T-bet Negative Forward | Biotin-TTAGCTAGTCGGCGCTATAGTTTTAAAACCTGATCGTAGATCGCTAGCTAGTA |
| T-bet Negative Reverse | TACTAGCTAGCGATCTACGATCAGGTTTTAAAACTATAGCGCCGACTAGCTAA |
| rs1006353 allele A Forward | Biotin-TGGAGCACCCATCCACATAGGCCCCAATGATAAGATAGCGTTAACCACCAGC |
| rs1006353 allele A Reverse | GCTGGTGGTTAACGCTATCTTATCATTGGGGCCTATGTGGATGGGTGCTCCA |
| rs1006353 allele G Forward | Biotin-TGGAGCACCCATCCACATAGGCCCCAGTGATAAGATAGCGTTAACCACCAGC |
| rs1006353 allele G Reverse | GCTGGTGGTTAACGCTATCTTATCACTGGGGCCTATGTGGATGGGTGCTCCA |
| rs1015290 allele A Forward | Biotin-GTGAGGAGGTGGGAGGAACATTCATGAGTGGGTAACCCTGTGACTAATCCAG |
| rs1015290 allele A Reverse | CTGGATTAGTCACAGGGTTACCCACTCATGAATGTTCCTCCCACCTCCTCAC |
| rs1015290 allele T Forward | Biotin-GTGAGGAGGTGGGAGGAACATTCATGTGTGGGTAACCCTGTGACTAATCCAG |
| rs1015290 allele T Reverse | CTGGATTAGTCACAGGGTTACCCACACATGAATGTTCCTCCCACCTCCTCAC |
| rs11135484 allele A Forward | Biotin-AGAAACAACACCAACCTCACACCCACATAACAGGATTAAAAGATAATGTGCA |
| rs11135484 allele A Reverse | TGCACATTATCTTTTAATCCTGTTATGTGGGTGTGAGGTTGGTGTTGTTTCT |
| rs11135484 allele G Forward | Biotin-AGAAACAACACCAACCTCACACCCACGTAACAGGATTAAAAGATAATGTGCA |
| rs11135484 allele G Reverse | TGCACATTATCTTTTAATCCTGTTACGTGGGTGTGAGGTTGGTGTTGTTTCT |
| rs13333528 allele C Forward | Biotin-ACAGCTGCTTGGTGACGTTCATGTGGCTGGTTTCATTACCACCTAACTCCCA |
| rs13333528 allele C Reverse | TGGGAGTTAGGTGGTAATGAAACCAGCCACATGAACGTCACCAAGCAGCTGT |
| rs13333528 allele T Forward | Biotin-ACAGCTGCTTGGTGACGTTCATGTGGTTGGTTTCATTACCACCTAACTCCCA |
| rs13333528 allele T Reverse | TGGGAGTTAGGTGGTAATGAAACCAACCACATGAACGTCACCAAGCAGCTGT |
| rs1420106 allele C Forward | Biotin-AACTCTTCTCTGACCAACCTCCTTGACGAGATAAAGTTTGTGGTTGGGTTTA |
| rs1420106 allele C Reverse | TAAACCCAACCACAAACTTTATCTCGTCAAGGAGGTTGGTCAGAGAAGAGTT |
| rs1420106 allele T Forward | Biotin-AACTCTTCTCTGACCAACCTCCTTGATGAGATAAAGTTTGTGGTTGGGTTTA |
| rs1420106 allele T Reverse | TAAACCCAACCACAAACTTTATCTCATCAAGGAGGTTGGTCAGAGAAGAGTT |
| rs1465321 allele A Forward | Biotin-ACCTGCCATCCAGGAGCTCCAAGGCTAGGGTTAACACTGAGGCCAACTGACC |
| rs1465321 allele A Reverse | GGTCAGTTGGCCTCAGTGTTAACCCTAGCCTTGGAGCTCCTGGATGGCAGGT |
| rs1465321 allele G Forward | Biotin-ACCTGCCATCCAGGAGCTCCAAGGCTGGGGTTAACACTGAGGCCAACTGACC |
| rs1465321 allele G Reverse | GGTCAGTTGGCCTCAGTGTTAACCCCAGCCTTGGAGCTCCTGGATGGCAGGT |
| rs2106346 allele A Forward | Biotin-AACGTATGTTGACAGACTTTTCAAATAATCATCTTTGTTATTACACCAAAAT |
| rs2106346 allele A Reverse | ATTTTGGTGTAATAACAAAGATGATTATTTGAAAAGTCTGTCAACATACGTT |
| rs2106346 allele C Forward | Biotin-AACGTATGTTGACAGACTTTTCAAATCATCATCTTTGTTATTACACCAAAAT |
| rs2106346 allele C Reverse | ATTTTGGTGTAATAACAAAGATGATGATTTGAAAAGTCTGTCAACATACGTT |
| rs2387397 allele C Forward | Biotin-ATGCAAATAAGAAGCTGTTTCAGTGTCTGCCCATCTGAGACGCTGACATAAA |
| rs2387397 allele C Reverse | TTTATGTCAGCGTCTCAGATGGGCAGACACTGAAACAGCTTCTTATTTGCAT |
| rs2387397 allele G Forward | Biotin-ATGCAAATAAGAAGCTGTTTCAGTGTGTGCCCATCTGAGACGCTGACATAAA |
| rs2387397 allele G Reverse | TTTATGTCAGCGTCTCAGATGGGCACACACTGAAACAGCTTCTTATTTGCAT |
| rs2703078 allele A Forward | Biotin-GCTATGACCACTGGCTCACATTTGCCACTTCAAGCCTCCTACAAGAAACATA |
| rs2703078 allele A Reverse | TATGTTTCTTGTAGGAGGCTTGAAGTGGCAAATGTGAGCCAGTGGTCATAGC |
| rs2703078 allele G Forward | Biotin-GCTATGACCACTGGCTCACATTTGCCGCTTCAAGCCTCCTACAAGAAACATA |
| rs2703078 allele G Reverse | TATGTTTCTTGTAGGAGGCTTGAAGCGGCAAATGTGAGCCAGTGGTCATAGC |
| rs2984920 allele A Forward | Biotin-GATACACGTCACAGCACACCAAGAAAAGGGGAACTTCCAGTGTCTGTGGTAA |
| rs2984920 allele A Reverse | TTACCACAGACACTGGAAGTTCCCCTTTTCTTGGTGTGCTGTGACGTGTATC |
| rs2984920 allele G Forward | Biotin-GATACACGTCACAGCACACCAAGAAAGGGGGAACTTCCAGTGTCTGTGGTAA |
| rs2984920 allele G Reverse | TTACCACAGACACTGGAAGTTCCCCCTTTCTTGGTGTGCTGTGACGTGTATC |
| rs3091310 allele C Forward | Biotin-TAAAATCATTGTTCAAATGAATGAATCAAGAGAAGTTTAAACCACTTTGGAC |
| rs3091310 allele C Reverse | GTCCAAAGTGGTTTAAACTTCTCTTGATTCATTCATTTGAACAATGATTTTA |
| rs3091310 allele G Forward | Biotin-TAAAATCATTGTTCAAATGAATGAATGAAGAGAAGTTTAAACCACTTTGGAC |
| rs3091310 allele G Reverse | GTCCAAAGTGGTTTAAACTTCTCTTCATTCATTCATTTGAACAATGATTTTA |
| rs5778 allele C Forward | Biotin-ACTCTTCCAGCCTCCCACATGATGGGCGGAAAAAGGCAAAAGCCCAGATTAA |
| rs5778 allele C Reverse | TTAATCTGGGCTTTTGCCTTTTTCCGCCCATCATGTGGGAGGCTGGAAGAGT |
| rs5778 allele T Forward | Biotin-ACTCTTCCAGCCTCCCACATGATGGGTGGAAAAAGGCAAAAGCCCAGATTAA |
| rs5778 allele T Reverse | TTAATCTGGGCTTTTGCCTTTTTCCACCCATCATGTGGGAGGCTGGAAGAGT |
| rs6784841 allele A Forward | Biotin-CAGCTGCAGTGTATGACTATACATCAATGACTCACACGGTGGGGGATCCCTC |
| rs6784841 allele A Reverse | GAGGGATCCCCCACCGTGTGAGTCATTGATGTATAGTCATACACTGCAGCTG |
| rs6784841 allele G Forward | Biotin-CAGCTGCAGTGTATGACTATACATCAGTGACTCACACGGTGGGGGATCCCTC |
| rs6784841 allele G Reverse | GAGGGATCCCCCACCGTGTGAGTCACTGATGTATAGTCATACACTGCAGCTG |
| rs743776 allele C Forward | Biotin-ATCCAAACTCTCAGTCTTGACCCACACGTCCTACAGGGACTGCCCCATCTTC |
| rs743776 allele C Reverse | GAAGATGGGGCAGTCCCTGTAGGACGTGTGGGTCAAGACTGAGAGTTTGGAT |
| rs743776 allele T Forward | Biotin-ATCCAAACTCTCAGTCTTGACCCACATGTCCTACAGGGACTGCCCCATCTTC |
| rs743776 allele T Reverse | GAAGATGGGGCAGTCCCTGTAGGACATGTGGGTCAAGACTGAGAGTTTGGAT |
| rs7441808 allele A Forward | Biotin-TATTTACCTTAGGGACTCCTCTGGGTATGTGAAGAATTCCCCTGTTTTGCTC |
| rs7441808 allele A Reverse | GAGCAAAACAGGGGAATTCTTCACATACCCAGAGGAGTCCCTAAGGTAAATA |
| rs7441808 allele G Forward | Biotin-TATTTACCTTAGGGACTCCTCTGGGTGTGTGAAGAATTCCCCTGTTTTGCTC |
| rs7441808 allele G Reverse | GAGCAAAACAGGGGAATTCTTCACACACCCAGAGGAGTCCCTAAGGTAAATA |
| rs8008961 allele C Forward | Biotin-CATATAAGATTGCCTCACATACTTGACTTTCATAGAAGCGGAAGCCATTGAC |
| rs8008961 allele C Reverse | GTCAATGGCTTCCGCTTCTATGAAAGTCAAGTATGTGAGGCAATCTTATATG |
| rs8008961 allele T Forward | Biotin-CATATAAGATTGCCTCACATACTTGATTTTCATAGAAGCGGAAGCCATTGAC |
| rs8008961 allele T Reverse | GTCAATGGCTTCCGCTTCTATGAAAATCAAGTATGTGAGGCAATCTTATATG |
| rs8062727 allele A Forward | Biotin-CCACAGGGGAAAAATGTGTGGTTGCCACCACTTCCTCTTATGGGGAAAGGAG |
| rs8062727 allele A Reverse | CTCCTTTCCCCATAAGAGGAAGTGGTGGCAACCACACATTTTTCCCCTGTGG |
| rs8062727 allele G Forward | Biotin-CCACAGGGGAAAAATGTGTGGTTGCCGCCACTTCCTCTTATGGGGAAAGGAG |
| rs8062727 allele G Reverse | CTCCTTTCCCCATAAGAGGAAGTGGCGGCAACCACACATTTTTCCCCTGTGG |
